# Optimization of Trepanning Patterns for Holes Ablated Using Nanosecond Pulse Laser in Al_2_O_3_ Ceramics Substrate

**DOI:** 10.3390/ma14143834

**Published:** 2021-07-09

**Authors:** Wanqin Zhao, Xuesong Mei

**Affiliations:** State Key Laboratory for Manufacturing Systems Engineering, Xi’an Jiaotong University, Xi’an 710049, China; linazhaolinazhao@foxmail.com

**Keywords:** filled spiral laser trepanning, multiple rings laser trepanning, hole, Al_2_O_3_, nanosecond pulse laser

## Abstract

Trepanning pattern is an important factor in laser hole machining, affecting both the hole quality and process efficiency. The influence of laser trepanning patterns on the hole ablating using nanosecond pulse laser in Al_2_O_3_ ceramics substrate was studied. Two laser trepanning patterns were evaluated, filled spiral trepanning and multiple rings trepanning, with the optimized laser machining parameters. In conjunction with the studies, the hole saturated taper and the saturated processing time were taken as the primary criteria for evaluation of the hole quality and the machining efficiency, respectively. Finally, the trepanning patterns were optimized aiming for the high hole quality; the process was based on the saturated hole tapers. The hole high qualities and machining efficiencies were obtained based on the saturated processing time, which was proven to have a great significance when using the nanosecond pulse laser to machine Al_2_O_3_ ceramics substrate.

## 1. Introduction

Alumina (Al_2_O_3_) ceramics have numerous valuable properties, including high thermal conductivity, high insulation and strength, and low thermal expansion coefficient. As such, Esmail et al. and Wang et al. expressed that they are often used as preferred materials for medium- and high-end electronic substrates in both the aerospace and information technology industries [1,2]. To achieve the high-density electronic system interconnection, it is usually required to process the holes located on the Al_2_O_3_ ceramics surface, making the double-sided lines connected based on THVF (Through Hole Via Filling). Therefore, high quality hole processing is very necessary, demonstrating none or little spatter, recast layer, burr, crack, and good roundness, etc. However, traditional machining methods have a high rejection rate and low processing efficiency due to the Al_2_O_3_ hardness and brittleness. Comparatively, laser drilling with high peak energy is among the best hole machining methods for ceramics substrates. Liu et al. and Zhao et al. summarized that the advantages for laser drilling are non-contact processing, lack of tool loss, high efficiency and precision, among others [3,4].

Considering the pulse duration, they mainly include millisecond laser, nanosecond laser, and ultrafast laser hole processing on electronic ceramic substrate. Firstly, many studies on millisecond pulse laser machining ceramics substrate are available; Mei et al. reviewed its processing mechanism is primarily the thermal effect with the thermal stress and soma chemical reactions [5], meaning that the processing quality is not ideal. Hanon et al. and Kacar et al. discussed the hole ablation by using millisecond pulse laser in Al_2_O_3_ ceramic, it can be seen from the top and profile figures of holes, there are dozens or even hundreds of micron surface spatters and side-wall recast [6,7]. In recent years, the short and ultra-short pulse laser technology was developed rapidly, and the related studies on its use for machining ceramic substrates were carried out. For example, Nedialkov et al. studied the N and O element contents in the heat-affected zone in detail, and the related chemical reaction mechanism [8,9]. Mutlu et al. found the average hole diameter and depth increase with the laser average power increasing [10]. Additionally, the ablation depth per pulse increased as the laser fluence increasing but slowly because of the plasma shielding effect [11]. Kong et al. reported the hole depth increased through adding the fluence, or minishing the repetition rate and scanning speed in both air and water [12]. For the ultrafast laser, Kim et al. [13], Li et al. [14], Chen et al. [15], Wang et al. [16], and Kim et al. [17] observed the laser drilling of Al_2_O_3_ and ALN ceramics using femtosecond pulses. Relationships between the processing parameters, the fluence/energy, number of pulses, focus position, and hole diameter/depth, ablation rate, were discussed. Besides, there is little or no spatters around the periphery of hole, a small amount of recast layer on side-wall, thinner and shorter cracks for short and ultrashort pulses of laser hole processing on ceramics [9,14,16,17]. In general, holes with higher quality can be produced for short and ultrafast laser than millisecond laser. However, the nanosecond laser is much cheaper than ultrafast laser, and generally offers the best cost-efficient. Therefore, the exploration on nanosecond laser ceramic hole machining has the most practical value.

Considering the hole ablation strategies, laser percussion drilling has been the most frequently used method for the general ablation of holes with a diameter below 100 μm. On the other hand, laser trepanning, being a percussion drilling process followed by a cutting procedure [18], is the most commonly employed process for larger diameter holes. Such as, Ashkenasi et al. reported the laser trepanning holes with the entrance diameters of 90 to 150 μm in stainless steel and ALN ceramic samples for industrial applications [19]. There are two main characteristics for laser trepanning. One, the sample upper surface coincides with the laser focal plane and remains unchanged, the other, the cutting paths or trepanning patterns are mainly filled with concentric circles, spiral and single or multiple rings and so on, the laser beam is scanned along with the designed trepanning patterns, removing the material, thus drilling holes. Furthermove, the trepanning patterns are very important. Thanking the Zhao et al. research for example, it had been found that the hole side-wall quality was better when the scanning direction was from the inside to the outside, and the ratio between the filling distance and the laser waist diameter was ranging from 2/9 and 1/3, for nanosecond laser trepanning filled with concentric circles patterns on ALN ceramics [20]. Compared with the most commonly used trepanning patterns concentric circles and spiral patterns, the research on single or multiple rings trepanning patterns is less. Wang et al. compared three different drilling methods, percussion drilling, single-ring cutting, and spiral trepanning, and found that the spiral trepanning method should be adopted for the film hole machining with the hole diameter larger than 100 µm in the K24 superalloy, for single-ring cutting, chip removal will be more and more difficult, and the surface chip will be heated repeated forming of heavy recasting and thermal effect areas [21]. Relatively, there are some advantages for multiple rings trepanning. Wang et al. [16] and Kim et al. [17] researched the variations in hole machining quality and the efficiency for both a single circle and three circle trepanning. The smaller tapers, improved hole circularity, and higher machining efficiency were observed in three circle trepanning, suggesting the great potential but less research. 

Based on the literature review, it can be concluded that the optimization of trepanning patterns is indeed effective, but very few studies were carried out on the subject. Thus, in this study, the effects of two laser trepanning patterns based on the UV nanosecond pulse, filled spiral trepanning and multiple rings trepanning, on the hole quality and efficiency in Al_2_O_3_ ceramics was studied. The optimization of trepanning patterns based on both machining quality and efficiency was carried out for the industry-standard Al_2_O_3_ ceramics with the common hole diameters.

## 2. Experiments and Materials

**Figure 1:**
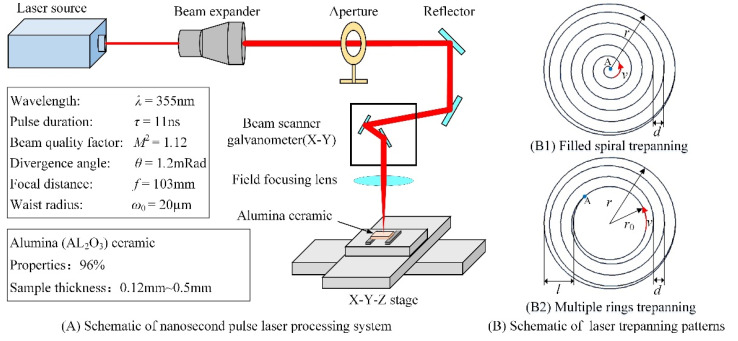
Schematics of nanosecond pulse laser processing system (**A**) and laser trepanning patters (**B**).

Figure 1A illustrates the nanosecond pulse laser drilling system, which includes the UV nanosecond pulse laser, the optical path transmission, the focusing system, the precise movement platform, and the control system. The las Diode Pumped Solid State (DPSS) Q-switched laser Fotia-355 with the laser wavelength λ of 355 nm was used in the experiment. The pulse duration *τ* was 11 ns, while the repetition rate *f* was 50 kHz. Moreover, the laser beam diameter was 1.1 mm, with a nominal beam ellipticity of 97.1%. The beam quality factor was *M*^2^ = 1.12, and its divergence angle was1.2 mRad. Generally, the beam energy conforms to the Gaussian distribution. Finally, the laser beam focusing spot diameter was approximately 20 μm and was transmitted and focused by the focusing field mirror with a focal length of 103 mm. 

Two laser trepanning patterns were studied in this article to obtain high quality and efficiency hole machining, filled spiral trepanning and multiple rings trepanning (see Figure 1B). Characteristic parameters for both patterns are the ex-radius *r*, which has a major influence on the inlet diameter, and gap fills *d*. In trepanning with multiple rings, specific parameters are the in-radius *r_0_* and the ring width *l*. It should also be noted that the laser beam path is taken from inside to outside, where *v* is the scanning speed. Once the first scan is finished, the laser beam moves to the starting position point A, and the second scan is started. Using that analogy, the scanning was completed *n* times.

Additionally, the experiments were carried out under ambient conditions. After the experiment, the scanning electron microscope (SEM), optical microscope (OM), and laser confocal microscope (LCM) were used to observe the hole morphologies and dimensions. Five holes were drilled using the same processing parameters, allowing to find the average value. The hole diameter was found through Equation (1):

(1) $$D=\frac{D_{max}-D_{min}}{2}$$

where *D* is the average hole diameter, *D_max_* and *D_min_* are the long and short axes of the hole center diameter, respectively.

The hole circularity *C* is equal to the ratio between the short *D_min_* and the long axis diameter *D_max_*, calculated by Equation (2) [22,23]:

(2) $$C=\frac{D_{min}}{D_{max}}$$

Additionally, the hole taper *α* is calculated as [24]:

(3) $$\alpha =tan^{-1}\left(\frac{D_{en}-D_{ex}}{2h}\right)$$

where *D_en_* and *D_ex_* are the hole inlet and outlet diameters, respectively, and *h* is the material thickness (hole depth).

In the experiment, 96% Al_2_O_3_ ceramics was used; test specimen length and width were 100 mm. Sample thicknesses were as follows: 0.12, 0.25, 0.38, and 0.5 mm, while the target machining inlet diameters were ranging from 100 to 1500 µm. It should be noted that those sample thicknesses and ablated inlet diameters are the most widely used as commercial ceramic electronic substrates. Lastly, before the experiment samples were cleaned using the ultrasonic for 10 min in combination with acetone and anhydrous ethanol. After the cleaning, samples were placed on a precision moving worktable.

## 3. The Hole Ablation Quality Comparison

### 3.1. Characteristic Hole Quality Evaluation Parameters

**Figure 2:**
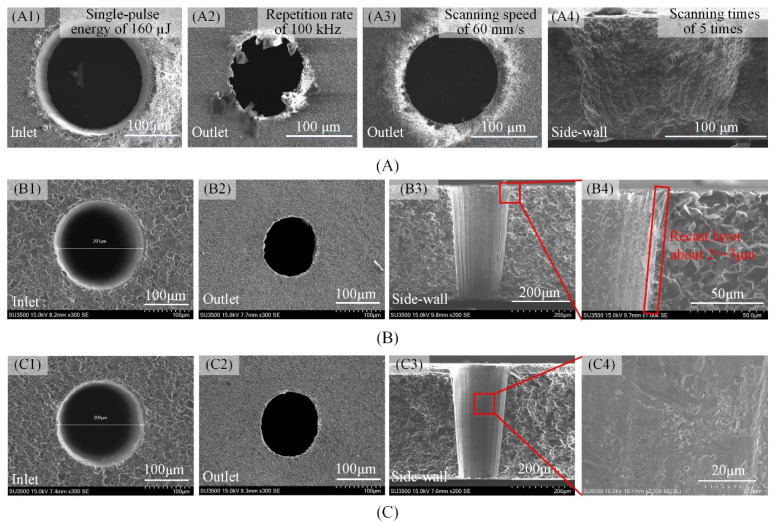
SEM of holes morphology ablated with optimal and non-optimal laser machining parameters in Al_2_O_3_ ceramics ((**B**,**C**), 0.38 mm thickness). (**A**) SEM of hole morphology ablated with non-optimal laser machining parameters, (**B**) SEM of hole morphology ablated with optimal laser machining parameters by filled spiral laser trepanning, (**C**) SEM of hole morphology ablated with optimal laser machining parameters by multiple rings laser trepanning.

The characteristic hole quality evaluation parameters ablated using the nanosecond pulse laser in Al_2_O_3_ ceramics are: the hole taper, the hole circularity (both the inlet and outlet circularity), the surface spatters around the hole inlet and outlet peripheries, the surface micro-crack, the heat-affected zone, and the recast layer on the side-wall surface, among others. In order to compare the two trepanning patterns and eliminate the influence of other machining parameters as much as possible, the optimal machining parameters were obtained on the basis of a large number of experiments. Figure 2A shows the SEM morphology of holes ablated with non-optimal laser machining parameters in Al_2_O_3_ ceramics ablated using nanosecond pulse laser. It can be seen that there are lots of spatters, burrs, and the heat-affected zone etc., because of high single pulse energy (Figure 2A1, single-pules energy of 160 µJ), or high repetition rate (Figure 2A2, repetition rate of 100 kHz), and slow scanning speed (Figure 2A3, sacnning speed of 60 mm/s) etc. For comparison, the SEM morphology of holes ablated with the optimized laser processing parameters are shown in Figure 2B,C, and the optimized laser machining parameters: the single-pulse energy of 120 μJ, the repetition rate of 50 kHz, 300 mm/s scanning speed, 4000 mm/s laser jump speed, and the scanning times of 300 times. Additionally, the fill spacing was 15 µm for filled spiral laser trepanning and 10 µm with the 40 µm wide ring for multiple rings laser trepanning. It is evident that there are no spatters, micro-cracks, and heat-affected zones; the hole outlet is elliptical and the contour edge is damaged by a small number of burrs. Furthermore, the side-wall hole is without cracks and is smooth, while the recast layer is only 2 to 3 µm thick. In other words, issues such as the spatter, micro-cracks, the heat-affected zones, and the recast layer, among others, could be practically eliminated by using the optimized laser machining parameters. Therefore, in this study, the characteristic hole quality evaluation parameters were primarily concerned with the hole taper, the outlet circularity, and the outlet contour edge burr.

The study at hand has shown that the optimized laser machining parameters for Al_2_O_3_ ceramics are the single-pulse energy of 120 μJ, the 50 kHz repetition rate, the 300 mm/s scanning speed, the laser jump speed of 4000 mm/s, while the scanning times were mainly determined by the sample thickness. Additionally, for the filled spiral laser trepanning the fill spacing was 15 µm, while it was µm 10 for multiple rings laser trepanning. Different ring widths were used; generally ring width was one-fifth of the inlet diameter when the inlet diameters were below 500 µm. The ring width was 100 µm, when the inlet diameters were between 1000 and 1500 µm.

### 3.2. Effect of Trepanning Patterns on the Hole Quality in Single-Thickness Al_2_O_3_ Ceramics

**Figure 3:**
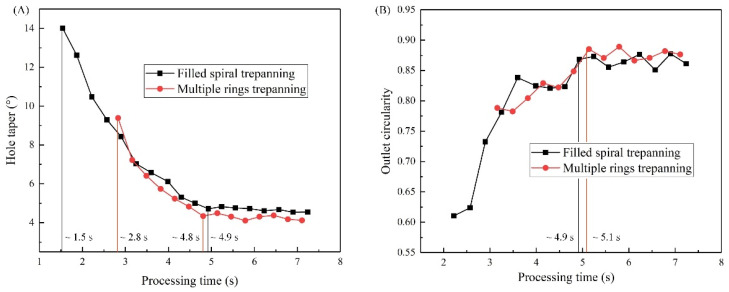
Hole taper and outlet circularity ablated with two trepanning patterns in Al_2_O_3_ ceramic (0.38 mm thickness), (**A**) Processing time versus hole taper, (**B**) Processing time versus outlet circularity.

The effect of trepanning patterns on the hole quality ablated using the nanosecond pulse laser with the optimal laser machining parameters was analyzed. The 0.38 mm thick Al_2_O_3_ ceramics was used as the test material. Comparisons between the hole tapers and outlet circularities are shown in Figure 3. As the processing time increases for both laser trepanning pattern, hole taper firstly decreases followed by the saturation; contrarily, hole outlets firstly increases, followed by the saturation.

**Figure 4:**
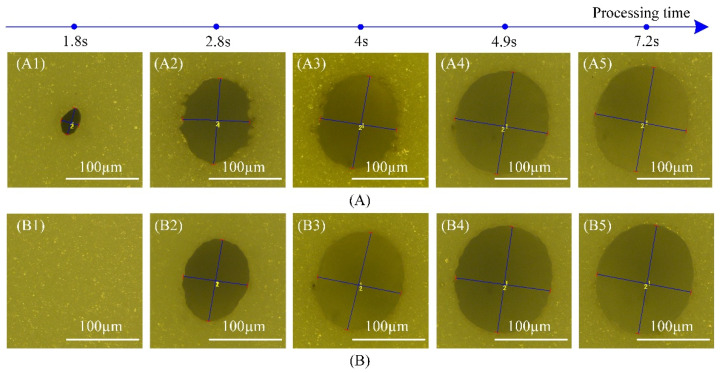
Outlets ablated using both laser trepanning patterns in 0.38 thick Al_2_O_3_ ceramics (figure obtained by OM), (**A**) OM of outlet ablated by filled spiral laser trepanning, (**A1**) The central region was penetrated, (**A2**,**A3**) The outlet was expanded, (**A4**) The outlet reached the saturated state, (**A5**) The outlet was modified, (**B**) OM of outlet ablated by multiple rings laser trepanning, (**B1**) Unshaped, (**B2**) The outlet formed, (**B3**) The outlet was expanded, (**B4**) The outlet reached the saturated state, (**B5**) The outlet was modified.

Furthermore, for filled spiral laser trepanning, it takes approximately 1.5 s to shape the through-hole, and roughly 4.9 s to saturate the hole taper with the 4.716° saturation taper. On the other hand, for multiple rings laser trepanning, roughly 2.8 s are needed for shaping and 4.8 s for the saturation with a 4.338° saturation taper, as shown in Figure 4A. In addition, it took about 4.9 s for the outlet circularity to reach the maximum values (approx. between 0.87 and 0.89) for filled spiral laser trepanning. In multiple rings laser trepanning, the times were slightly longer, around 5.1 s. In short, the material penetration property in Al_2_O_3_ ceramics is stronger for filled spiral laser trepanning, as evidenced by the shorter through-hole shape time. At the same time, the multiple rings laser trepanning strategy may produce smaller saturated hole taper and larger outlet circularity, indicating that it has more efficient expanding and material removal abilities.

Figure 4 shows the OM views of outlet morphologies ablated using a nanosecond pulse laser in 0.38 mm thick Al_2_O_3_ ceramic. The outlet size was very small when shaping the new through-hole using filled spiral laser trepanning (Figure 4A1). As the processing time increased, the hole outlet was expanded into an oval shape containing some contour edge burrs (Figure 4A2,A3). With the further progress of the processing time, the outlet reached the saturated size displaying a smooth profile, a larger circularity, and practically no burrs (Figure 4A4). Then, the outlet was modified with the increase in processing time; however, the outlet quality was similar to the one observed in the saturated state (Figure 4A4,A5). In other words, the subsequent pulses had a rather limited effect. 

Different from the filled spiral laser trepanning, the hole was shaped slowly with a large oval. On the other hand, it was smooth and only a few burrs were found on the outlet while using multiple rings laser trepanning (Figure 4B2). The subsequent pulses have shown a good ability to both expand and smooth the outlet (Figure 4B3). Eventually, the outlet size and circularity became saturated (Figure 4B4). The same behavior was observed for both laser trepanning methods; subsequent pulses had a limited effect (Figure 4B4,B5).

**Figure 5:**
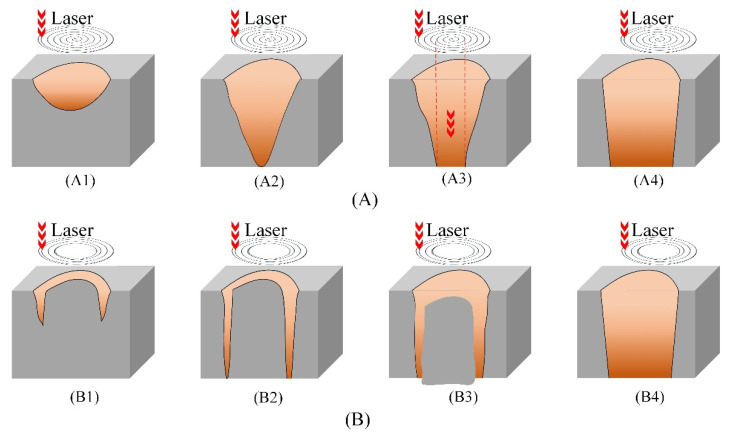
Schematic diagram of filled spiral laser trepanning and multiple rings laser trepanning machining mechanisms, (**A**) Filled spiral laser trepanning, (**A1**) Material removed layer by layer, (**A2**) Material penetrated in the center, (**A3**) Outlet expansion, (**A4**) Outlet has reached the saturated state, (**B**) Multiple rings laser trepanning, (**B1**) Ring-shaped removal of the material, (**B2**) Ring cutting gap was formed, (**B3**) Material at the center fell off from the outlet, (**B4**) The outlet reached the saturated state.

The difference in outlet morphologies was mainly due to machining mechanism variations between the trepanning patterns, as shown in Figure 5. For filled spiral laser trepanning (see Figure 5A), pattern tracks completely cover the material, removing it layer-by-layer (Figure 5A1). With the increase in processing time, the material located in the hole center is completely penetrated first, due to the high energy concentration, shaping the through-hole (Figure 5A2). In the next step, the outlet is expanded using only the laser energy stemming from the outer scanning track ring, while the laser energy from the inner scanning track ring penetrated through the hole center without effect (Figure 5A3). Finally, the outlet reached the saturated state (Figure 5A4). 

For comparison, in multiple rings laser trepanning shown in Figure 5B, laser-ablated along the outer scanning track ring in a manner similar to cutting. Only the material located at the hole edge was removed layer-by-layer (Figure 5B1). When the ring cutting gap formed, the material at the hole center fell off from the outlet due to actions of gravity and impact force, shaping the through-hole (Figure 5B2,B3). Then, the outlet finally reached the saturated state (Figure 5B4). Since the energy concentration in the outer scanning track ring was sufficient, the outlet edge was modified consistently, resulting in better outlet circularity and a lower amount of contour edge burrs. Additionally, the outlet size was slightly larger than the one ablated by filled spiral laser trepanning in the same processing moment. 

Therefore, based on the above-presented research, it can be concluded that the smaller the hole saturated taper, the larger the outlet circularity and the better the outlet profile morphology. In other words, the higher the hole quality. Hence, the saturated taper hole was taken as the main criterion to evaluate the hole machining quality.

### 3.3. Effect of Trepanning Patterns on Hole Quality in Al_2_O_3_ Ceramics 

**Figure 6:**
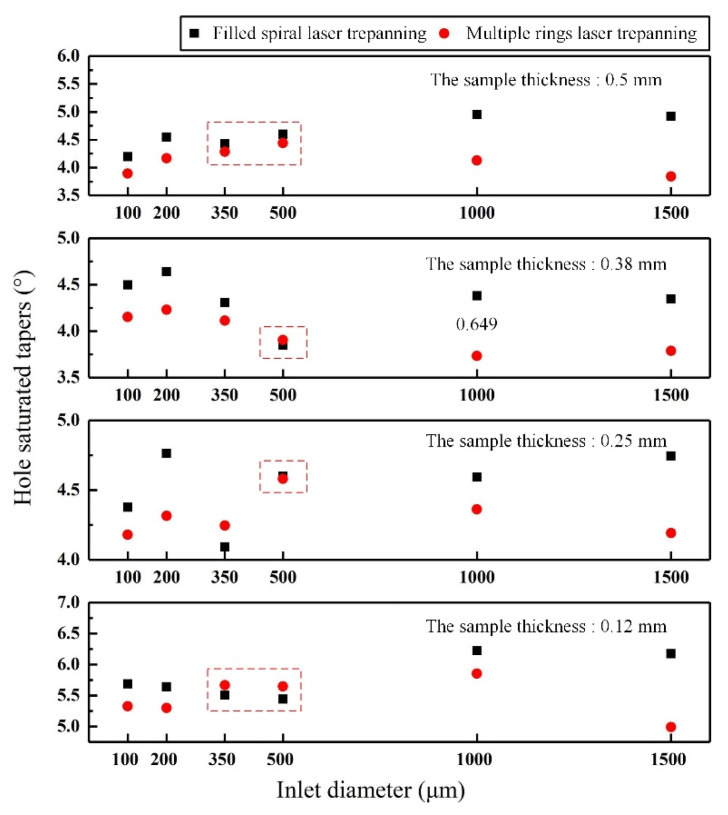
Saturated hole tapers ablated in Al_2_O_3_ ceramics of various thicknesses.

Figure 6 shows the saturated hole tapers ablated using nanosecond pulse laser in Al_2_O_3_ ceramics. The thicknesses ranged between 0.12 and 0.5 mm, while the inlet diameters were between 100 and 1500 μm. When the inlet diameters were below 200 μm and above 1000 μm, all the saturated hole tapers processed using multiple rings laser trepanning were smaller than their filled spiral laser trepanning counterparts, as shown in Figure 6. In other words, the multiple rings laser trepanning had a better processing quality. When the inlet diameters were between 350 and 500 μm, the saturated hole tapers processed by both trepanning patterns were relatively similar, as shown using the red dotted boxes in Figure 6. The processing quality for both trepanning patterns was rather similar in that case. The reason for such behavior is that, when there are in very small inlet diameter, the laser drilling using multiple rings trepanning avoids the energy waste caused by its concentration in the central area of the hole (Figure 5A3), resulting in a higher material removal rate and a smaller hole taper. When the inlet diameter is very large, the material in the hole center is cut off (Figure 5B3), resulting in high machining efficiency and a small hole taper.

**Figure 7:**
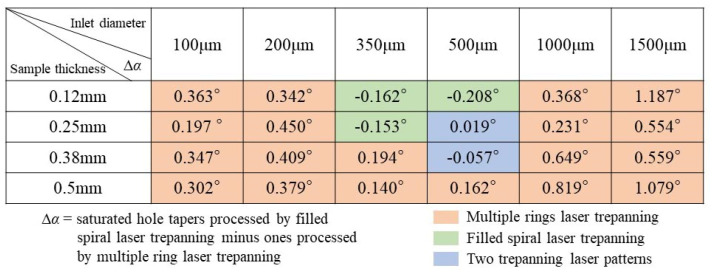
Optimization of trepanning patterns for hole quality based on the hole saturated tapers.

Figure 7 shows differences in values for the saturated hole tapers ablated by two trepanning patterns. The ∆*α* values were equal to the difference between the saturated hole tapers processed by filled spiral laser trepanning minus the ones processed by multiple rings laser trepanning. In other words, when the ∆*α* values are above zero, the saturated hole tapers processed using the filled spiral laser trepanning are larger than their counterparts made using multiple rings laser trepanning. Consequently, multiple rings laser trepanning should be selected for the smaller saturated hole taper due to its better hole quality (the orange area in Figure 7). On the other hand, filled spiral laser trepanning should be employed when ∆α is a sub-zero value (the green area in Figure 7). It should also be noted that, when the samples were 0.25 and 0.38 mm thick and the inlet diameter was 500 µm, the ∆*α* differences 0.02 and −0.06, respectively. At this point, each of the laser trepanning patterns can be selected, as the machining quality regarding the hole saturated taper will practically be the same (the blue area in Figure 7).

## 4. Comparison of Hole Quality and Machining Efficiency

**Figure 8:**
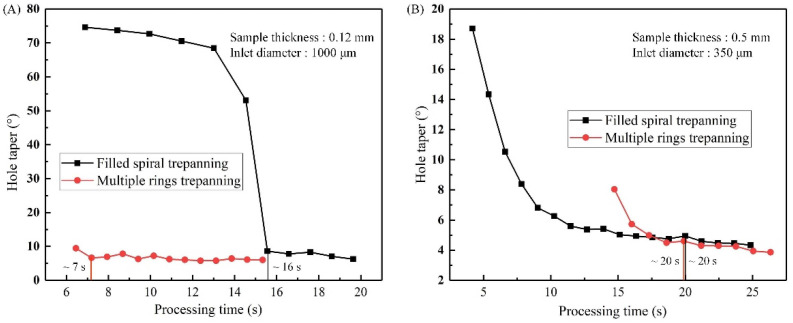
Hole taper vs the processing time ablated using both trepanning patterns, (**A**) Processing time versus hole taper with sample thickness of 0.12 mm and hole diameter of 1000 µm, (**B**) Processing time versus hole taper with sample thickness of 0.5 mm and hole diameter of 350 µm.

The hole machining efficiencies obtained by two laser trepanning patterns using the optimized laser machining parameters were analyzed and compared. The processing time needed to reach the saturated state (saturated processing time) was used as the evaluation criterion. The hole quality has reached the best values for the results shown in Section 3.1. Moreover, Figure 8 shows the relationship between the hole taper and the processing time for both trepanning patterns. The sample thickness was 0.12 mm and the inlet diameter was 1000 μm in Figure 8A, and they were 0.5 mm and 350 μm in Figure 8B. It is evident that the saturated processing times ablated by filled spiral laser trepanning and the multiple rings laser trepanning were approximately 16 and 7 s (see Figure 8A), and roughly 20 s (Figure 8B). For the hole processing shown in Figure 8A, saturated processing times (hole machining efficiencies) based on multiple rings laser trepanning were about twice the filled spiral laser trepanning values. On the other hand, in Figure 8B, the multiple rings laser trepanning time is almost the same with the filled spiral laser trepanning time.

**Figure 9:**
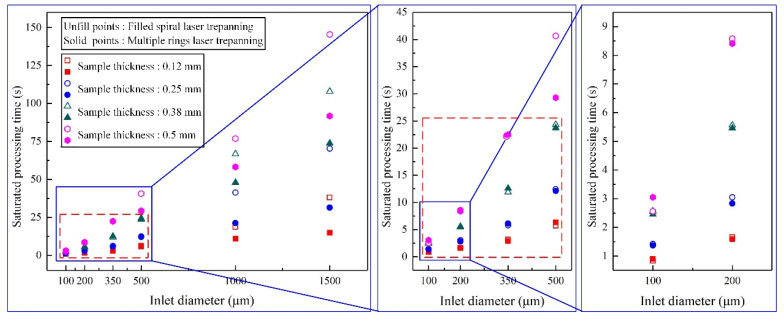
Saturated hole processing times for both trepanning patterns.

Figure 9 shows the saturated hole processing times ablated using nanosecond pulse laser in Al_2_O_3_ ceramics. When the inlet diameters were above 1000 μm with 0.12 to 0.38 mm thick samples, and the above 500 μm in 0.5 mm thick samples, the saturated hole processing times were longer for filled spiral laser trepanning (Figure 9). In other words, the multiple rings laser trepanning had higher efficiency. When the inlet diameters were below 350 μm for all the considered sample thicknesses, and when the inlet diameters were below 500 μm in 0.12 to 0.38 mm thick samples, the saturated hole processing times were similar for both methods, as shown in the red dotted boxes (Figure 9). Thus, in that case, the processing efficiencies were rather similar.

**Figure 10:**
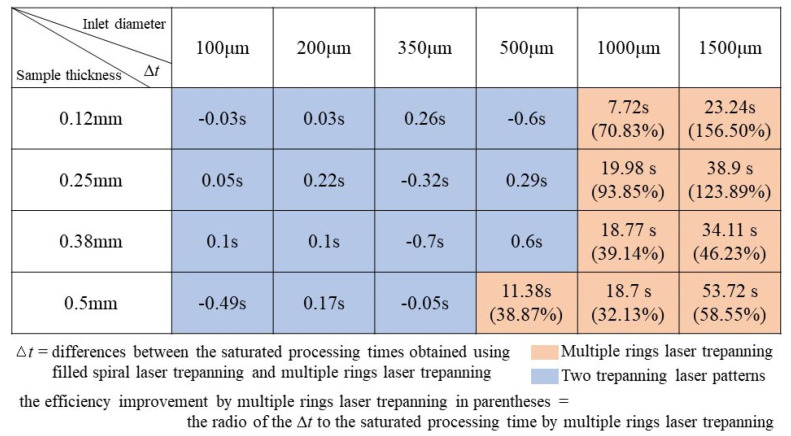
Optimization of trepanning patterns for high-quality holes and the machining efficiency based on the saturated processing time.

The data in Figure 10 show the differences in the saturated processing times ∆*t*, which are defined as the differences between the saturated processing times obtained using the filled spiral laser trepanning and the multiple rings laser trepanning. When the ∆*t* above zero, the saturated processing time needed by filled spiral laser trepanning is longer, meaning that the multiple rings trepanning laser should be selected (the orange area in Figure 10). Taking the minimum and maximum ∆*t* values (the orange area of Figure 10) as examples; the 7.72 s for 0.12 mm thickness and 1000 μm inlet diameter, and 53.72 s for 0.5 mm thickness and 1500 μm inlet diameter. The saturated processing time for the minimum ∆*t* by filled spiral laser trepanning and multiple rings laser trepanning are 18.62 and 10.9 s, respectively. For the maximum, they are 145.47 and 91.75 s, respectively. Compared to the machining efficiency by filled spiral laser trepanning, the efficiency improvement achieved by the use of multiple rings trepanning is 70.83% and 58.55%, respectively. The efficiency improvement obtained by multiple rings trepanning ranges from 32.13% to 156.50% (see Figure 10). For the remaining combinations, these values are below 1 s (the blue area in Figure 10), meaning that there is a minor difference in the processing efficiencies between two laser trepanning patterns. Therefore, both methods can be used effectively.

## 5. Conclusions

The influence of two laser trepanning patterns used in the UV nanosecond pulse filled spiral trepanning and multiple rings trepanning, on the hole quality and efficiency was studied. The test specimens were made of Al_2_O_3_ ceramics of industry-standard substrate thicknesses (between 0.12 and 0.5 mm), while the common hole diameters were between 100 and 1500 µm. The main criteria used to evaluate the hole quality and the machining efficiency were the saturated hole taper and the saturated processing time, respectively.

The research has shown that, for obtaining the high hole quality, the inlet diameters were below 200 μm or above 1000 μm for all the studied sample thicknesses, furthermore, the inlet diameter was 350 μm for the sample thicknesses between 0.38 and 0.5 mm and 500 μm for the 0.5 mm sample thickness, the results were satisfying for the multiple rings trepanning.

In filled spiral trepanning, when the inlet diameter was 350 μm for sample thicknesses ranging from 0.12 to 0.25 mm, and 500 μm for the 0.12 mm sample thickness. The results were considered to be satisfying. Furthermore, when the inlet diameter was 500 μm, sample thicknesses were between 0.25 and 0.38 mm, justifying the use of both laser trepanning patterns.

Regarding the hole quality and processing efficiency, when the inlet diameters were above 1000 μm and the sample thicknesses between 0.12 and 0.38 mm, and the inlet diameters above 500 μm in combination with the sample thickness of 0.5 mm, multiple rings trepanning provided good results. The efficiency improvement when using multiple rings trepanning was thus ranging from 32.13% to 156.50%. Furthermore, when the inlet diameters were below 350 μm and the sample thicknesses ranged between 0.12 and 0.5 mm, as well as the inlet diameters below 500 μm with the sample thicknesses between 0.12 and 0.38 mm, both laser trepanning patters provided good results.

## Data Availability

Exclude this statement.
